# Establishment and validation of nomograms to predict the overall survival and cancer-specific survival for non-metastatic bladder cancer patients: A large population-based cohort study and external validation

**DOI:** 10.1097/MD.0000000000037492

**Published:** 2024-03-15

**Authors:** Shan Li, Jinkui Wang, Zhaoxia Zhang, Yuzhou Wu, Zhenyu Liu, Zhikang Yin, Junhong Liu, Dawei He

**Affiliations:** a Department of Urology, Children’s Hospital of Chongqing Medical University, Chongqing, China; b Chongqing Key Laboratory of Children Urogenital Development and Tissue Engineering, Chongqing, China; c China International Science and Technology Cooperation base of Child Development and Critical Disorders, National Clinical Research Center for Child Health and Disorders, Ministry of Education Key Laboratory of Child Development and Disorders, Chongqing Key Laboratory of Pediatrics, Children’s Hospital of Chongqing Medical University, Chongqing, China; d Department of Urology, The First Affiliated Hospital of Chongqing Medical University, Chongqing, China.

**Keywords:** bladder cancer, CSS, nomogram, OS, prognostic prediction, SEER

## Abstract

This study aimed to develop nomograms to accurately predict the overall survival (OS) and cancer-specific survival (CSS) of non-metastatic bladder cancer (BC) patients. Clinicopathological information of 260,412 non-metastatic BC patients was downloaded from the Surveillance, Epidemiology, and End Results (SEER) database from 2000 to 2020. LASSO method and Cox proportional hazard regression analysis were utilized to discover the independent risk factors, which were used to develop nomograms. The accuracy and discrimination of models were tested by the consistency index (C-index), the area under the subject operating characteristic curve (AUC) and the calibration curve. Decision curve analysis (DCA) was used to test the clinical value of nomograms compared with the TNM staging system. Nomograms predicting OS and CSS were constructed after identifying independent prognostic factors. The C-index of the training, internal validation and external validation cohort for OS was 0.722 (95%CI: 0.720–0.724), 0.723 (95%CI: 0.721–0.725) and 0.744 (95%CI: 0.677–0.811). The C-index of the training, internal validation and external validation cohort for CSS was 0.794 (95%CI: 0.792–0.796), 0.793 (95%CI: 0.789–0.797) and 0.879 (95%CI: 0.814–0.944). The AUC and the calibration curves showed good accuracy and discriminability. The DCA showed favorable clinical potential value of nomograms. Kaplan–Meier curve and log-rank test uncovered statistically significance survival difference between high- and low-risk groups. We developed nomograms to predict OS and CSS for non-metastatic BC patients. The models have been internally and externally validated with accuracy and discrimination and can assist clinicians to make better clinical decisions.

## 1. Introduction

Bladder cancer (BC) ranks as the sixth most common malignancy in the United States, with estimated 82,290 new cases and 16,710 death cases in the United States in 2023.^[1]^ Among all malignancies for male patients, the morbidity of BC ranked fourth and the mortality of BC ranked tenth.^[1]^ About 3.0% of new cancer patients were diagnosed with BC, and 2.1% of cancer deaths were due to BC.^[2]^ Based on the depth of malignancies infiltrating the bladder wall, tumors at stage Tis, Ta, and T1 are clinically named non-muscle-invasive bladder cancer (NMIBC), and tumors at stage T2, T3, and T4 are clinically named muscle-invasive bladder cancer (MIBC). NMIBC Patients with grade 1, 2, and 3 tumors have a favorable prognosis with a 5-year cancer-specific mortality of 0.5%, 1.7%, and 6.8%, respectively.^[3]^ However, about 25% of BC patients have grown into MIBC at diagnosis,^[4]^ and up to 40% to 50% of patients with NMIBC will finally develop into MIBC, which resulted in a bad prognosis for BC patients. Radical cystectomy (RC) has been considered the main treatment method for MIBC, but the oncological outcome of 5-year overall survival rate is only about 50% after RC.^[5]^ In addition, invasive surgical procedures with urine flow diversions can worsen the life quality and may harm the mental health of BC patients. RC surgery may cause some elderly patients to become bedridden, making them fail to maintain good urination, renal function, electrolyte balance, and well general condition.^[6]^ So, it is important to construct an efficient prediction model for BC patients to predict their prognosis precisely, especially for patients with MIBC.

Nowadays, there are appropriate treatment options for different pathology and stages of BC, such as transurethral resection of bladder tumors (TURBT), bladder immunotherapy with Bacillus Calmette - Guerin (BCG), intravesical chemotherapy, and radiation therapy. For patients with non-metastatic BC, their treatment regimen is a comprehensive treatment with surgery as the main therapy, supplemented by radiotherapy and chemotherapy. Staging of primary tumor-regional lymph nodes-distant metastasis (TNM) is a considerate tool to evaluate prognosis and develop a comprehensive treatment for non-metastatic BC. Nevertheless, with the same TNM stage and other similar clinicopathological features, patients with non-metastatic BC may have different oncological outcomes during clinical practices. Some patients die prematurely after surgical treatment, while other patients still survive or even live longer than expected.

The 8th TNM staging system was officially released in January 2017 by American Joint Committee on Cancer and Union for International Cancer Control (UICC).^[7]^ Currently, the TNM staging system is considered to be the most significant prognostic tool for the recurrence and survival of BC patients, providing guidance and help for the selection of surgical treatment and postoperative supplementary treatment.^[8]^ The new TNM staging system was more detailed and constructed based on more patient data, but it still has some limitations. For example, some details in the database revising the staging system are missing, causing many descriptions unable to be analyzed. Moreover, some vital clinical pathological factors are absent in the TNM staging system, including race, age, gender, surgery type, histological grade, marriage, and physical status.^[9–12]^ As one of factors affecting the prognosis, TNM staging is only a classification according to the basic lesion characteristic of tumors, which cannot dominate the prognosis prediction and treatment choice of BC patients. Hence, a more trustworthy and precise prognostic model for non-metastatic BC patients is urgently required.

Nomogram is a concise graphical mathematical model, enabling researchers to forecast the occurrence of an outcome event by producing a single numerical estimate on the basis of clinical and pathological factors.^[13]^ Nomogram is extensively applied in developing treatment plans and predicting survival for tumor patients.^[14–16]^ To the extent of our knowledge, no nomogram has been established to predict the clinical outcome of non-metastatic BC patients. Based on the clinical pathological parameters collected from the Surveillance Epidemiology and End Results (SEER) database, we constructed nomograms to evaluate the prognosis of non-metastatic BC patients, which can offer assistance for urologists to tailor treatment plan for every patient.

## 2. Material and methods

### 2.1. Data source and data extraction

Raw clinic data were collected from the SEER project (http://seer.cancer.gov/) of the National Cancer Institute from 2000 to 2020 on August 7, 2023. The SEER database contains approximately 28% of Americans and involves 18 tumor registries in the United States.^[17]^ Ethical approval and patient consent were not demanded since the patient information from the SEER database is anonymously disclosed. To establish the external validation cohort, we collected clinic data of non-metastatic BC patients in Department of Urology, The First Affiliated Hospital of Chongqing Medical University from 2010 to 2023. The study was conducted according to the guidelines of the Declaration of Helsinki and was approved by the Ethics Committee of The First Affiliated Hospital of Chongqing Medical University (protocol code: K2023-338). Informed consent was obtained from all subjects involved in this study. The clinical information of external validation cohort is listed in Supplementary Table 1, http://links.lww.com/MD/L909.

**Figure 1.**
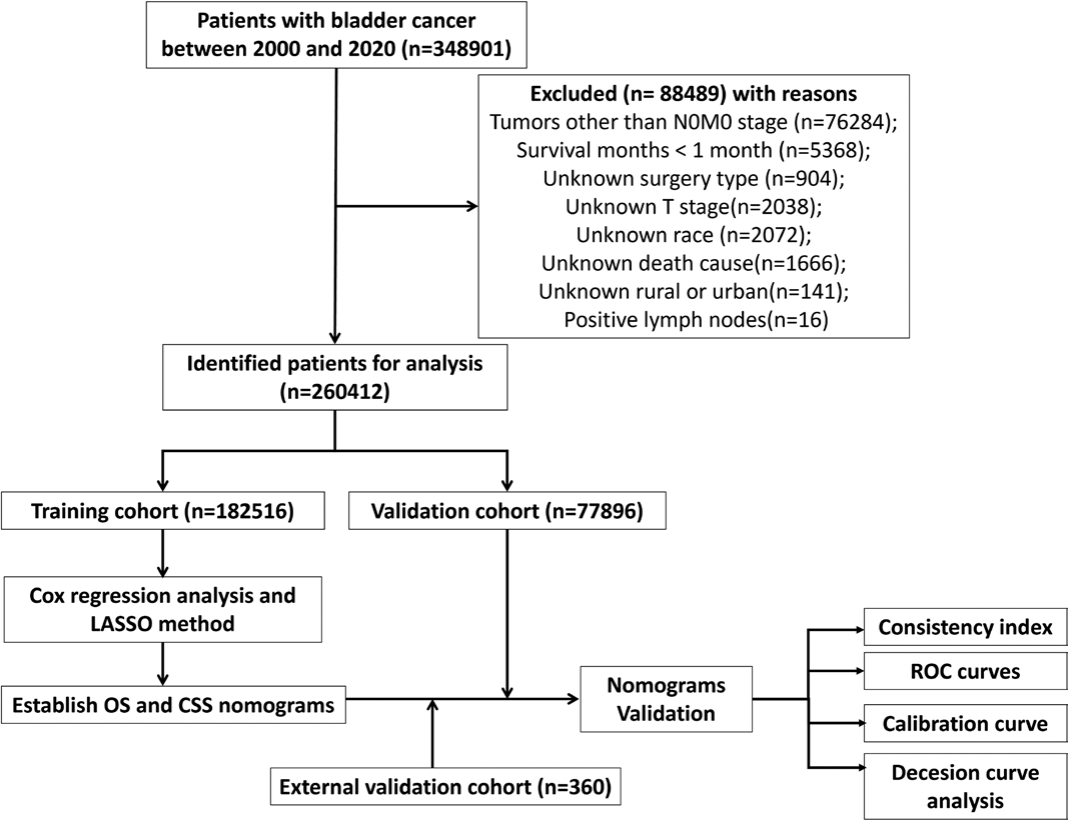
The flowchart for screening patients in this study.

Extracted data involves demographic information (age, gender, race, marital status, household location), tumor characteristics (grade, TNM stage, tumor size, tumor primary location, pathology, laterality), therapy method (surgery, lymph node surgery, radiotherapy, chemotherapy) and follow-up information (overall and cancer-specific survival status, survival months). The selection criteria are non-metastatic tumors at stage N0M0; the first diagnosis was registered as bladder site record (C67.0-C67.9) according to the Third Edition of International Classification of Diseases for Oncology (ICD-O-3). The exclusion criteria are non-primary tumor; unknown surgical method; unknown TNM stage; unknown pathological differentiation; unknown location of the tumor; survival duration less than 1 month; patients without positive diagnostic confirmation. Our flowchart for screening patients is displayed in Figure 1.

Two continuous variables, age and tumor size, were transformed to categorical variables. The age was classified into four groups according to accepted cutoff values: <40, 40 to 59, 60 to 79,≥80 years. The tumor size was classified into seven categories: 0-2.0 cm, 2.1 to 4.0 cm, 4.1 to 6.0 cm, 6.1 to 8.0 cm, 8.1 to 10.0 cm, >10.0 cm, unknown. Based on literature review, the primary site of tumors was split into four groups: “Lateral wall of bladder,” “Bladder base,” “Urachus/Dome of bladder,” “Overlapping lesion of bladder.” According to SEER code, the histology was divided into five groups (“Transitional cell carcinoma,” “Adenocarcinoma,” “Squamous cell carcinoma,” “Epithelial carcinoma,” “Other”), and the surgery types were split into five categories (“No Surgery,” “Local tumor destruction/excision,” “Partial cystectomy,” “Complete cystectomy,” “Complete cystectomy with pelvic exenteration”). The overall survival (OS) focuses on the time from the diagnosis to the death or the last follow-up. The cancer-specific survival (CSS) was defined as the period from the diagnosis to the death from BC.

### 2.2. Nomogram construction and validation

We randomly allocated patients to a training cohort (70%) and a validation cohort (30%). In the training cohort, univariate and multivariate Cox regression were conducted to identify the independent risk factors and calculate the hazard ratio (HR) and the 95% confidence interval (CI). The least absolute shrinkage and selection operator (LASSO) was utilized to select ultimate risk factors and avoid collinearity. Incorporating the independent risk factors selected by LASSO and Cox method, we construct nomograms to forecast the 3-, 5-, and 8-year OS and CSS of non-metastatic BC patients. Based on the validation cohort, we utilize the calibration curve, the consistency index (C-index), and the area under the receiver operating characteristic curve (AUC), to validate the efficiency of nomograms. The accuracy of nomograms was checked by the calibration curve based on 1000 bootstrap resampling, which was conducted to investigate the relevance between the observed value and the actual value. The closer the curve is to the diagonal, the more accurate the nomogram is. We utilize C-index and AUC to evaluate the precision and discriminability of the nomograms.

We appraise the application value and the clinical benefits of the models by decision curve analysis (DCA), which is a new algorithm estimating the net benefit under every risk threshold.^[18]^ We computed the risk scores of every patient from the nomograms and determined the best cutoff values using the receiver operating characteristic (ROC) curve. According to the cutoff values, we split the patients into a low-risk and a high-risk group based on the risk scores of every patient. And we utilized the Kaplan–Meier curve and log-rank test to compare the OS and CSS of patients between two risk groups. Besides, we analyzed the survival differences between different surgeries in two risk groups.

### 2.3. Statistical analysis

Chi-squared test was utilized to compare the categorical variables in baseline characteristics. All statistics were performed using R software (version 4.2.2; http://www.Rproject.org). The R packages “rms,” “foreign,” “survminer,” “DynNom,” “nomogramformula,” “survival,” “survivalROC,” “RMS,” “pec,” and “ggDCA” were utilized to construct and validate the nomograms. All tests were two-sided, and *P* value < 0.05 was considered statistically significant.

## 3. Results

### 3.1. Clinicopathological characteristics

We enrolled 260,412 non-metastatic BC patients between 2000 and 2020, which were randomly allocated to the training cohort (N = 182,516) and the validation cohort (N = 77,896). The mean age of all patients was 70.9 ± 11.3 years, and most patients were male (76.6%), white (90.6%), married (60.6%), and urban residents (86.8%). The T stage comprised T1/Tis/Ta (82.9%), T2 (11.8%), T3 (3.24%) and T4 (2.04%). Tumors in the bladder wall (72.8%) were the most in terms of primary site. Transitional cell carcinoma (96.5%) was the dominating histology. Most tumors were not paired sites (98.5%) for laterality. Most patients underwent surgery of local tumor destruction/excision (86.3%). And 80.3% of patients were not treated by lymph node surgery. Then 81.8% of patients underwent chemotherapy, and 3.72% underwent radiotherapy. The mean survival time of both cohorts was 76.4 ± 63.1 months. The clinicopathological data are displayed in Table 1.

**Table 1:** Clinicopathological characteristics of non-metastatic BC patients retrieved from SEER database.

	Overall N = 260412	Training cohort N = 182137	Validation cohort N = 78275	*P* value
Age:				.862
<40	2666 (1.02%)	1852 (1.02%)	814 (1.04%)	
40–59	39538 (15.2%)	27599 (15.2%)	11939 (15.3%)	
60–79	148233 (56.9%)	103730 (57.0%)	44503 (56.9%)	
>=80	69975 (26.9%)	48956 (26.9%)	21019 (26.9%)	
Age (continuous):	70.9 (11.3)	70.9 (11.3)	70.9 (11.3)	.548
Race:				.952
White	236027 (90.6%)	165061 (90.6%)	70966 (90.7%)	
Black	13012 (5.00%)	9109 (5.00%)	3903 (4.99%)	
Other	11373 (4.37%)	7967 (4.37%)	3406 (4.35%)	
Sex:				.085
Male	199490 (76.6%)	139698 (76.7%)	59792 (76.4%)	
Female	60922 (23.4%)	42439 (23.3%)	18483 (23.6%)	
Marital status:				.070
Married	157938 (60.6%)	110456 (60.6%)	47482 (60.7%)	
Unmarried	47005 (18.1%)	32887 (18.1%)	14118 (18.0%)	
Widowed	37435 (14.4%)	26047 (14.3%)	11388 (14.5%)	
Unknown	18034 (6.93%)	12747 (7.00%)	5287 (6.75%)	
Household location:				.852
Rural	34469 (13.2%)	24093 (13.2%)	10376 (13.3%)	
Urban	225943 (86.8%)	158044 (86.8%)	67899 (86.7%)	
Tumor primary site:				.648
Bladder wall	189477 (72.8%)	132479 (72.7%)	56998 (72.8%)	
Bladder base	35265 (13.5%)	24760 (13.6%)	10505 (13.4%)	
Urachus/Dome of bladder	9590 (3.68%)	6702 (3.68%)	2888 (3.69%)	
Overlapping lesion	26080 (10.0%)	18196 (9.99%)	7884 (10.1%)	
Histology:				.636
Adenocarcinoma	1609 (0.62%)	1117 (0.61%)	492 (0.63%)	
Epithelial carcinoma	3104 (1.19%)	2193 (1.20%)	911 (1.16%)	
Other	1070 (0.41%)	759 (0.42%)	311 (0.40%)	
Squamous cell carcinoma	3410 (1.31%)	2357 (1.29%)	1053 (1.35%)	
Transitional cell carcinoma	251219 (96.5%)	175711 (96.5%)	75508 (96.5%)	
Grade:				.652
I	29820 (11.5%)	20897 (11.5%)	8923 (11.4%)	
II	62799 (24.1%)	43965 (24.1%)	18834 (24.1%)	
III	40046 (15.4%)	28063 (15.4%)	11983 (15.3%)	
IV	52980 (20.3%)	36917 (20.3%)	16063 (20.5%)	
Unknown	74767 (28.7%)	52295 (28.7%)	22472 (28.7%)	
Laterality:				.793
Bilateral	3979 (1.53%)	2791 (1.53%)	1188 (1.52%)	
Lateral	256433 (98.5%)	179346 (98.5%)	77087 (98.5%)	
T Stage:				.523
T1/Tis/Ta	215907 (82.9%)	151092 (83.0%)	64815 (82.8%)	
T2	30753 (11.8%)	21502 (11.8%)	9251 (11.8%)	
T3	8429 (3.24%)	5840 (3.21%)	2589 (3.31%)	
T4	5323 (2.04%)	3703 (2.03%)	1620 (2.07%)	
Tumor size:				.375
0–2.0 cm	35104 (13.5%)	24453 (13.4%)	10651 (13.6%)	
2.1–4.0 cm	39329 (15.1%)	27681 (15.2%)	11648 (14.9%)	
4.1–6.0 cm	20519 (7.88%)	14338 (7.87%)	6181 (7.90%)	
6.1–8.0 cm	4806 (1.85%)	3378 (1.85%)	1428 (1.82%)	
8.1–10.0 cm	1739 (0.67%)	1223 (0.67%)	516 (0.66%)	
>10.0 cm	3401 (1.31%)	2353 (1.29%)	1048 (1.34%)	
Unknown	155514 (59.7%)	108711 (59.7%)	46803 (59.8%)	
Surgery type:				.291
No surgery	13054 (5.01%)	9051 (4.97%)	4003 (5.11%)	
Local tumor destruction/excision	224760 (86.3%)	157351 (86.4%)	67409 (86.1%)	
Partial cystectomy	3163 (1.21%)	2200 (1.21%)	963 (1.23%)	
Complete cystectomy	12148 (4.66%)	8427 (4.63%)	3721 (4.75%)	
Complete cystectomy with pelvic exenteration	7287 (2.80%)	5108 (2.80%)	2179 (2.78%)	
Surgery other sites:				.995
None	222534 (85.5%)	155638 (85.5%)	66896 (85.5%)	
Yes	3180 (1.22%)	2223 (1.22%)	957 (1.22%)	
Unknown	34698 (13.3%)	24276 (13.3%)	10422 (13.3%)	
Lymph nodes surgery:				.997
None	209101 (80.3%)	146256 (80.3%)	62845 (80.3%)	
Regional lymph nodes removed	16446 (6.32%)	11499 (6.31%)	4947 (6.32%)	
Unknown	34865 (13.4%)	24382 (13.4%)	10483 (13.4%)	
Chemotherapy:				.810
None/unknown	213028 (81.8%)	149018 (81.8%)	64010 (81.8%)	
Yes	47384 (18.2%)	33119 (18.2%)	14265 (18.2%)	
Radiation:				.375
None/unknown	250730 (96.3%)	175405 (96.3%)	75325 (96.2%)	
Yes	9682 (3.72%)	6732 (3.70%)	2950 (3.77%)	
Neoadjuvant or adjuvant chemotherapy:				.024
None	114567 (44.0%)	79933 (43.9%)	34634 (44.2%)	
Chemotherapy before surgery	20802 (7.99%)	14467 (7.94%)	6335 (8.09%)	
Chemotherapy after surgery	36260 (13.9%)	25608 (14.1%)	10652 (13.6%)	
Before and after	1249 (0.48%)	872 (0.48%)	377 (0.48%)	
Unknown	87534 (33.6%)	61257 (33.6%)	26277 (33.6%)	
Neoadjuvant or adjuvant radiotherapy:				.883
None	251212 (96.5%)	175721 (96.5%)	75491 (96.4%)	
radiation before surgery	144 (0.06%)	105 (0.06%)	39 (0.05%)	
radiation after surgery	8941 (3.43%)	6230 (3.42%)	2711 (3.46%)	
before and after	65 (0.02%)	47 (0.03%)	18 (0.02%)	
Unknown	50 (0.02%)	34 (0.02%)	16 (0.02%)	
Survival mo	76.4 (63.1)	76.3 (63.0)	76.5 (63.2)	.441
Vital status:				.106
Alive	121368 (46.6%)	85076 (46.7%)	36292 (46.4%)	
Dead	139044 (53.4%)	97061 (53.3%)	41983 (53.6%)	
Cancer specific death:				.132
Not cancer specific death	211577 (81.2%)	148119 (81.3%)	63458 (81.1%)	
Death due to bladder cancer	48835 (18.8%)	34018 (18.7%)	14817 (18.9%)	

BC = bladder cancer, SEER = surveillance, epidemiology, and end results.

### 3.2. Nomograms development

**Figure 2.**
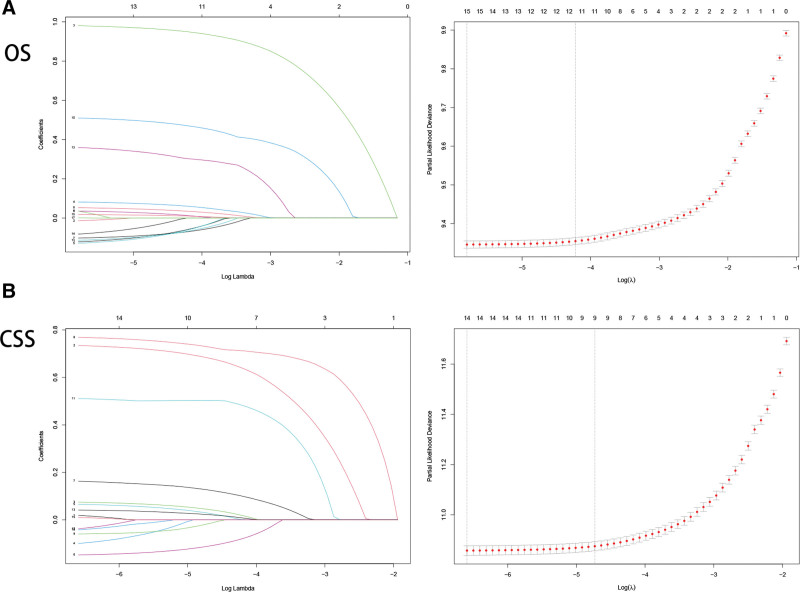
The LASSO regression method utilized to screen prognostic factors for OS (A) and CSS (B). CSS = cancer specific survival, LASSO = the least absolute shrinkage and selection operator, OS = overall survival.

**Figure 3.**
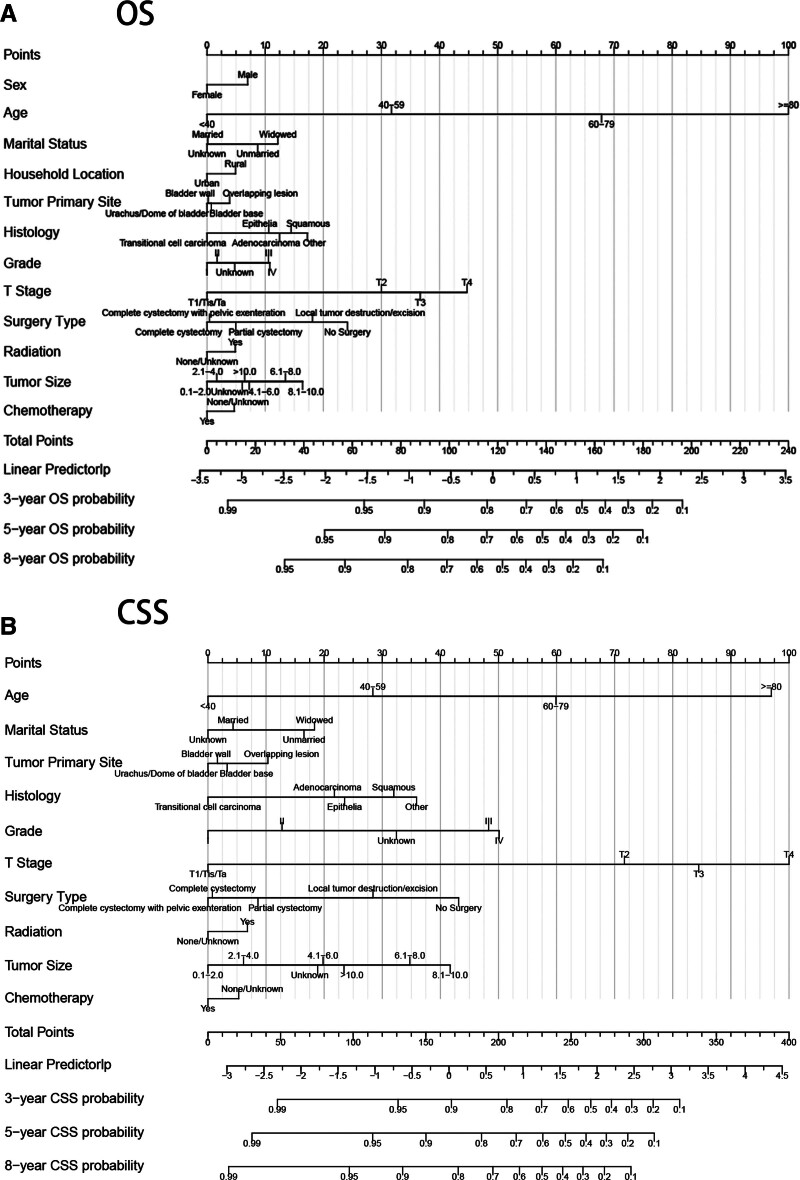
Nomograms for predicting 3-, 5-, 8-yr OS and CSS in non-metastatic BC patients. (A) The nomogram for predicting OS of non-metastatic BC patients. (B) The nomogram for predicting CSS of non-metastatic BC patients. BC = bladder cancer, CSS = cancer specific survival, OS = overall survival.

In training cohort, univariate and multivariate Cox regression was utilized to select independent risk factors influencing OS (Table 2) and CSS (Table 3). Using the log (λ) values chosen by one standard error of the minimum criteria, LASSO method (Fig. 2) screened the variables with non-zero coefficients to achieve simple and interpretable models. After screening, we discovered that age, sex, marital status, household location, tumor primary site, histology, grade, T stage, surgery type, radiation and tumor size were independent risk factors for OS. And age, marital status, tumor primary site, histology, grade, T stage, surgery type, radiation and tumor size were independent risk factors for CSS. Based on experience and guidelines, chemotherapy was vital in prognosis prediction of non-metastatic BC patients, which was also entered into Cox proportional hazard models to build nomograms. Finally, we involved 12 and 10 variables to establish nomograms to predict 3-, 5-, and 8-year OS and CSS in non-metastatic BC patients (Fig. 3).

**Table 2:** Univariate and multivariate analyses of OS in training cohort.

Characteristics	Univariate Cox			Multivariate Cox		
	HR	*P*	CI	HR	*P*	CI
Age:						
<40						
40–59	2.61	<.001	2.27–2.99	2.506	<.001	2.182–2.878
60–79	7.56	<.001	6.6–8.67	7.165	<.001	6.249–8.215
>=80	21.14	<.001	18.44–24.24	18.164	<.001	15.836–20.833
Race:						
White						
Black	1.15	<.001	1.12–1.19	1.179	<.001	1.145–1.213
Other	0.84	<.001	0.81–0.87	0.842	<.001	0.814–0.871
Sex:						
Male						
Female	0.95	<.001	0.94–0.96	0.811	<.001	0.798–0.825
Marital Status:						
Married						
Unmarried	1.05	<.001	1.03–1.07	1.272	<.001	1.249–1.295
Widowed	2.12	<.001	2.09–2.16	1.406	<.001	1.381–1.432
Unknown	0.98	.07	0.95–1	0.996	.767	0.969–1.024
Household location:						
Rural						
Urban	0.9	<.001	0.88–0.91	0.873	<.001	0.857–0.889
Tumor primary site:						
Bladder wall						
Bladder base	0.95	<.001	0.93–0.97	1.012	.222	0.993–1.031
Urachus/Dome of bladder	1.18	<.001	1.15–1.22	0.991	.607	0.959–1.025
Overlapping lesion	1.29	<.001	1.26–1.32	1.106	<.001	1.084–1.129
Histology:						
Adenocarcinoma						
Epithelial carcinoma	1.03	.441	0.95–1.13	0.961	.375	0.88–1.049
Other	1.07	.246	0.96–1.19	1.144	.018	1.024–1.277
Squamous cell carcinoma	1.03	.535	0.94–1.12	1.069	.125	0.982–1.165
Transitional cell carcinoma	0.58	<.001	0.54–0.62	0.707	<.001	0.658–0.76
Grade:						
I						
II	1.09	<.001	1.06–1.11	1.054	<.001	1.031–1.078
III	1.85	<.001	1.8–1.89	1.365	<.001	1.333–1.399
IV	1.9	<.001	1.86–1.94	1.406	<.001	1.373–1.44
Unknown	1.31	<.001	1.28–1.34	1.172	<.001	1.143–1.201
Laterality:						
Bilateral						
Lateral	1.15	0	1.08–1.22	1.077	.021	1.011–1.147
T Stage:						
T1/Tis/Ta						
T2	2.52	<.001	2.48–2.57	2.368	<.001	2.317-2.421
T3	2.33	<.001	2.26-2.41	2.802	<.001	2.697-2.911
T4	3.67	<.001	3.54-3.8	3.572	<.001	3.432-3.717
Tumor size:						
0-2.0						
2.1-4.0	1.2	<.001	1.17-1.23	1.053	<.001	1.025–1.082
4.1–6.0	1.65	<.001	1.6–1.7	1.24	<.001	1.203–1.279
6.1–8.0	2.1	<.001	2–2.2	1.5	<.001	1.428–1.577
8.1–10.0	2.42	<.001	2.25–2.61	1.64	<.001	1.52–1.769
>10.0	1.31	<.001	1.23–1.39	1.193	<.001	1.124–1.267
Unknown	1.31	<.001	1.29–1.34	1.17	<.001	1.144–1.196
Surgery type:						
No surgery						
Local tumor destruction/excision	0.85	<.001	0.83–0.88	0.875	<.001	0.849–0.902
Partial cystectomy	1.08	.014	1.02–1.15	0.655	<.001	0.614–0.699
Complete cystectomy	1.09	<.001	1.05–1.13	0.617	<.001	0.586–0.649
Complete cystectomy with pelvic exenteration	1.03	.213	0.98–1.08	0.651	<.001	0.612–0.693
Surgery other sites:						
None						
Yes	1.34	<.001	1.27–1.42	0.999	.983	0.944–1.058
Unknown	0.99	.172	0.97–1.01	0.914	.198	0.797–1.048
Lymph nodes surgery:						
None						
Regional lymph nodes removed	1.1	<.001	1.07–1.13	0.749	<.001	0.717–0.782
Unknown	0.99	.365	0.98–1.01	1.086	.238	0.947–1.244
Chemotherapy:						
None/Unknown						
Yes	1.07	<.001	1.05–1.09	1.037	.006	1.011–1.064
Radiation:						
None/Unknown						
Yes	3.23	<.001	3.15–3.32	1.371	<.001	1.215–1.546
Neoadjuvant or Adjuvant Chemotherapy:						
None						
Chemotherapy before surgery	0.71	<.001	0.69–0.73	0.734	<.001	0.705–0.763
Chemotherapy after surgery	0.95	<.001	0.93–0.97	0.805	<.001	0.785–0.827
Before and after	1.01	.794	0.91–1.13	0.895	.049	0.801–1
Unknown	0.96	<.001	0.94–0.97	0.979	.014	0.962–0.996
Neoadjuvant or Adjuvant Radiotherapy:						
None						
Radiation before surgery	2.04	<.001	1.64–2.53	0.882	.319	0.688–1.13
Radiation after surgery	3.21	<.001	3.12–3.3	0.801	<.001	0.708–0.905
Before and after	1.98	<.001	1.4–2.81	0.535	.001	0.37–0.772
Unknown	1.97	<.001	1.35–2.88	0.779	.212	0.526–1.153

HR = hazard ratio, OS = overall survival.

**Table 3:** Univariate and multivariate analyses of CSS in training cohort.

Characteristics	Univariate Cox			Multivariate Cox		
	HR	*P*	CI	HR	*P*	CI
Age:						
<40						
40–59	2.19	<.001	1.8–2.66	1.86	<.001	1.529–2.261
60–79	4.26	<.001	3.51–5.16	3.485	<.001	2.872–4.228
>=80	10.31	<.001	8.5–12.51	7.252	<.001	5.973–8.805
Race:						
White						
Black	1.51	<.001	1.44–1.57	1.346	<.001	1.289–1.405
Other	0.93	.014	0.88–0.99	0.893	<.001	0.845–0.943
Sex:						
Male						
Female	1.14	<.001	1.11–1.17	0.997	.799	0.971–1.023
Marital status:						
Married						
Unmarried	1.17	<.001	1.13–1.2	1.249	<.001	1.213–1.286
Widowed	2.08	<.001	2.02–2.13	1.297	<.001	1.259–1.337
Unknown	0.86	<.001	0.82–0.9	0.911	<.001	0.867–0.957
Household location:						
Rural						
Urban	0.92	<.001	0.9–0.95	0.896	<.001	0.869–0.924
Tumor primary site:						
Bladder wall						
Bladder base	0.95	.005	0.92–0.99	1.03	.073	0.997–1.064
Urachus/Dome of bladder	1.31	<.001	1.24–1.38	0.968	.231	0.917–1.021
Overlapping lesion	1.61	<.001	1.56–1.66	1.182	<.001	1.145–1.22
Histology:						
Adenocarcinoma						
Epithelial carcinoma	1.08	.212	0.96–1.21	1.061	.333	0.941–1.195
Other	1.25	.002	1.08–1.45	1.326	<.001	1.147–1.534
Squamous cell carcinoma	1.1	.117	0.98–1.23	1.252	<.001	1.114–1.407
Transitional cell carcinoma	0.39	<.001	0.35–0.43	0.673	<.001	0.609–0.743
Grade:						
I						
II	1.34	<.001	1.28–1.41	1.293	<.001	1.228–1.362
III	4.44	<.001	4.23–4.66	2.587	<.001	2.459-2.72
IV	4.69	<.001	4.47-4.92	2.726	<.001	2.592-2.866
Unknown	2.31	<.001	2.19-2.43	1.919	<.001	1.821-2.022
Laterality:						
Bilateral						
Lateral	1.17	.003	1.05-1.29	1.058	.279	0.955-1.171
T Stage:						
T1/Tis/Ta						
T2	5.28	<.001	5.15–5.41	4.008	<.001	3.886–4.133
T3	5.36	<.001	5.15–5.57	5.084	<.001	4.833–5.348
T4	8.74	<.001	8.36–9.13	6.865	<.001	6.528–7.22
Tumor Size:						
0–2.0						
2.1–4.0	1.49	<.001	1.42–1.56	1.125	<.001	1.072–1.181
4.1–6.0	2.6	<.001	2.48–2.74	1.469	<.001	1.396–1.547
6.1–8.0	4.24	<.001	3.95–4.54	1.98	<.001	1.846–2.125
8.1–10.0	5.13	<.001	4.65–5.67	2.283	<.001	2.065–2.523
>10.0	1.92	<.001	1.75–2.12	1.538	<.001	1.398–1.693
Unknown	1.69	<.001	1.63–1.76	1.43	<.001	1.372–1.49
Surgery type:						
No surgery						
Local tumor destruction/excision	0.79	<.001	0.75–0.83	0.785	<.001	0.746–0.827
Partial cystectomy	1.59	<.001	1.46–1.73	0.6	<.001	0.545–0.66
Complete cystectomy	1.75	<.001	1.65–1.85	0.568	<.001	0.527–0.613
Complete cystectomy with pelvic exenteration	1.74	<.001	1.62–1.86	0.58	<.001	0.532–0.633
Surgery other sites:						
None						
Yes	2	<.001	1.86–2.16	1.005	.891	0.93–1.087
Unknown	0.95	<.001	0.92–0.97	0.871	.23	0.694–1.092
Lymph Nodes Surgery:						
None						
Regional lymph nodes removed	1.87	<.001	1.8–1.93	0.726	<.001	0.684–0.77
Unknown	0.99	.629	0.96–1.02	1.052	.657	0.84–1.319
Chemotherapy:						
None/Unknown						
Yes	1.43	<.001	1.39–1.46	1.075	<.001	1.034–1.118
Radiation:						
None/Unknown						
Yes	5.17	<.001	4.99–5.36	1.19	.028	1.019–1.39
Neoadjuvant or Adjuvant Chemotherapy:						
None						
Chemotherapy before surgery	0.66	<.001	0.63–0.7	0.638	<.001	0.598–0.68
Chemotherapy after surgery	1.25	<.001	1.21–1.29	0.859	<.001	0.824–0.894
Before and after	1.87	<.001	1.64–2.12	0.983	.808	0.86–1.125
Unknown	0.98	.222	0.96–1.01	0.979	.161	0.951–1.008
Neoadjuvant or Adjuvant Radiotherapy:						
None						
Radiation before surgery	3.75	<.001	2.88–4.89	1.133	.426	0.833–1.539
Radiation after surgery	5.1	<.001	4.91–5.29	0.904	.209	0.772–1.058
Before and after	3.56	<.001	2.32–5.46	0.609	.033	0.386–0.961
Unknown	4.04	<.001	2.57–6.33	1.129	.615	0.704–1.811

CSS = cancer specific survival, HR = hazard ratio.

### 3.3. Nomogram validation

**Figure 4.**
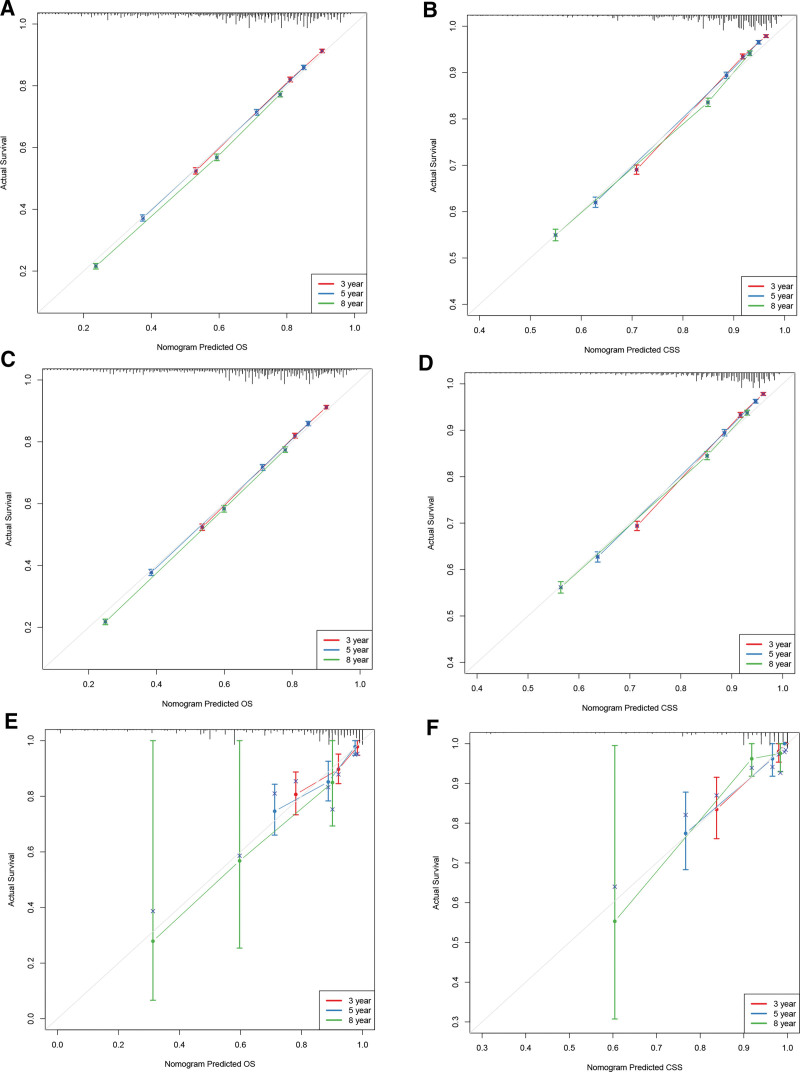
Calibration curve of the nomograms for predicting 3-, 5-, 8-yr OS and CSS in non-metastatic BC patients. Calibration curve of the nomograms for predicting 3-, 5-, 8-yr OS in the training cohort (A), internal validation cohort (C), and external validation cohort (E). Calibration curve of the nomograms for predicting 3-, 5-, 8-yr CSS in the training cohort (B), internal validation cohort (D), and external validation cohort (F). The horizontal axis is the predicted value in the nomogram, and the vertical axis is the observed value. BC = bladder cancer, CSS = cancer specific survival, OS = overall survival.

**Figure 5.**
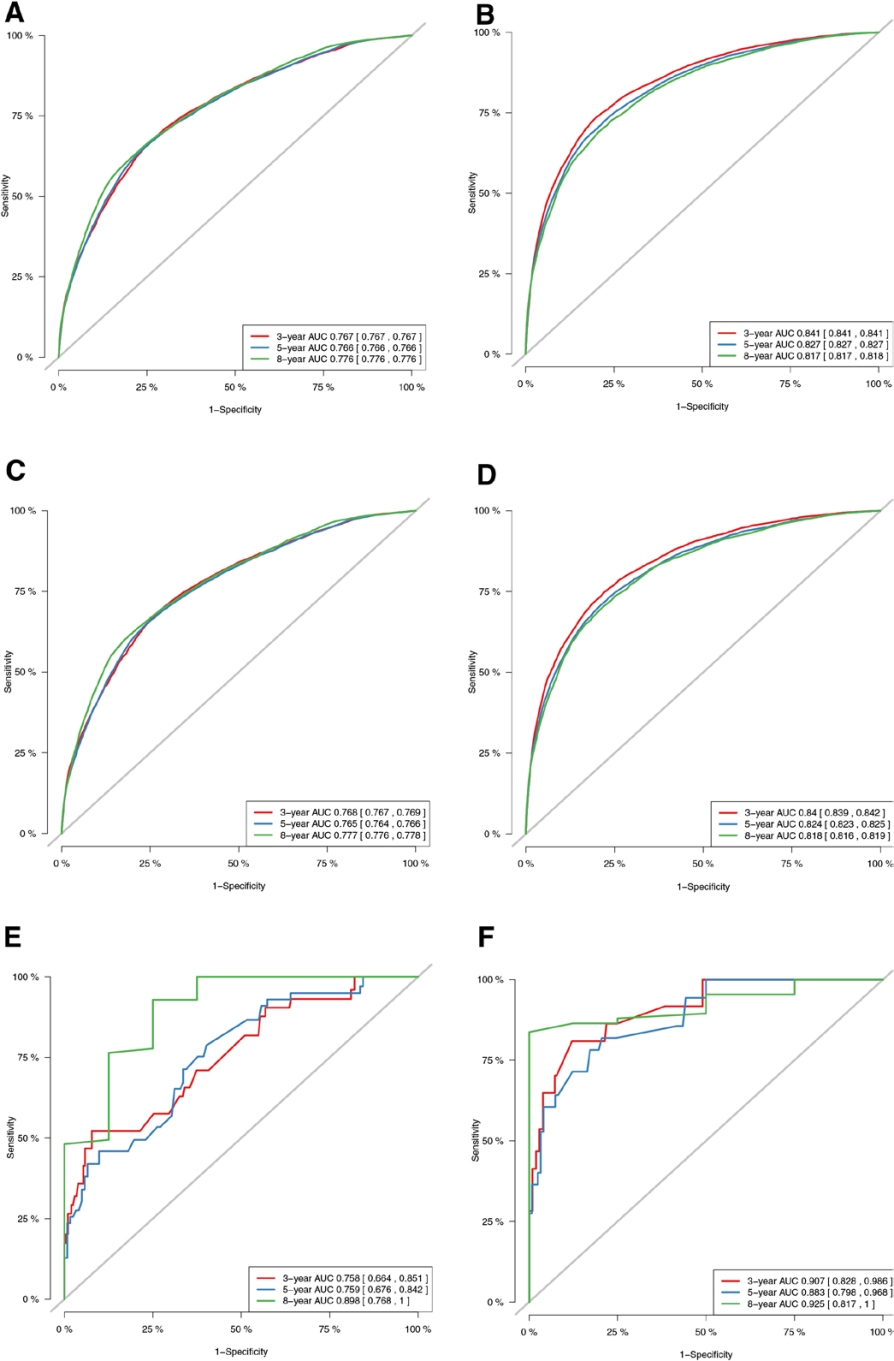
AUC for predicting 3-, 5-, and 8-yr CSS and OS in non-metastatic BC patients. ROC of the nomograms for predicting 3-, 5-, 8-yr OS in the training cohort (A), internal validation cohort (C), and external validation cohort (E). ROC of the nomograms for predicting 3-, 5-, 8-yr CSS in the training cohort (B), internal validation cohort (D), and external validation cohort (F). AUC = area under the receiver operating characteristic curve, BC = bladder cancer, CSS = cancer specific survival, OS = overall survival, ROC = receiver operating characteristic.

The C-index of the training and internal validation cohort for OS was 0.722 (95%CI: 0.720–0.724) and 0.723 (95%CI: 0.721–0.725). The C-index of the training and internal validation cohort for CSS was 0.794 (95%CI: 0.792–0.796) and 0.793 (95%CI: 0.789–0.797). As for external validation cohort in Chongqing (n = 364), the C-index for OS was 0.744 (95%CI: 0.677–0.811), and the C-index for CSS was 0.879 (95%CI: 0.814–0.944). The C-index displayed that the models are discriminative. Based on the training, internal validation and external validation cohort, the calibration curve displays that the predicted value for OS and CSS has great alignments with the observed value (Fig. 4). It indicates that the prediction of the models is accurate. With the AUC more than 0.7, the ROC curves reveals that the nomograms have favorable discrimination and accuracy (Fig. 5).

### 3.4. Clinical application of the nomograms

**Figure 6.**
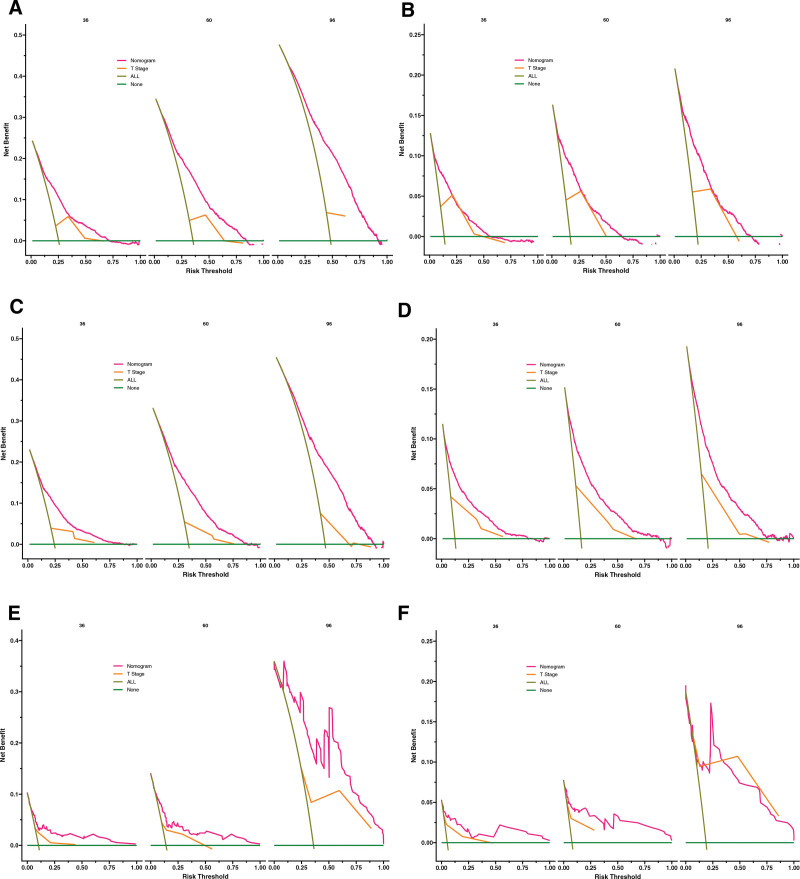
DCA of the nomograms for predicting OS and CSS. The nomogram for OS at 3, 5, 8-yr showed a better clinical application value than the T staging system in the training (A), internal validation (C) and external validation cohorts (E). The nomogram for CSS at 3-, 5-, 8-yr showed a better clinic application value than the T staging system in the training (B), internal validation (D) and external validation cohorts (F). CSS = cancer specific survival, DCA = decision curves analysis, OS = overall survival.

**Figure 7.**
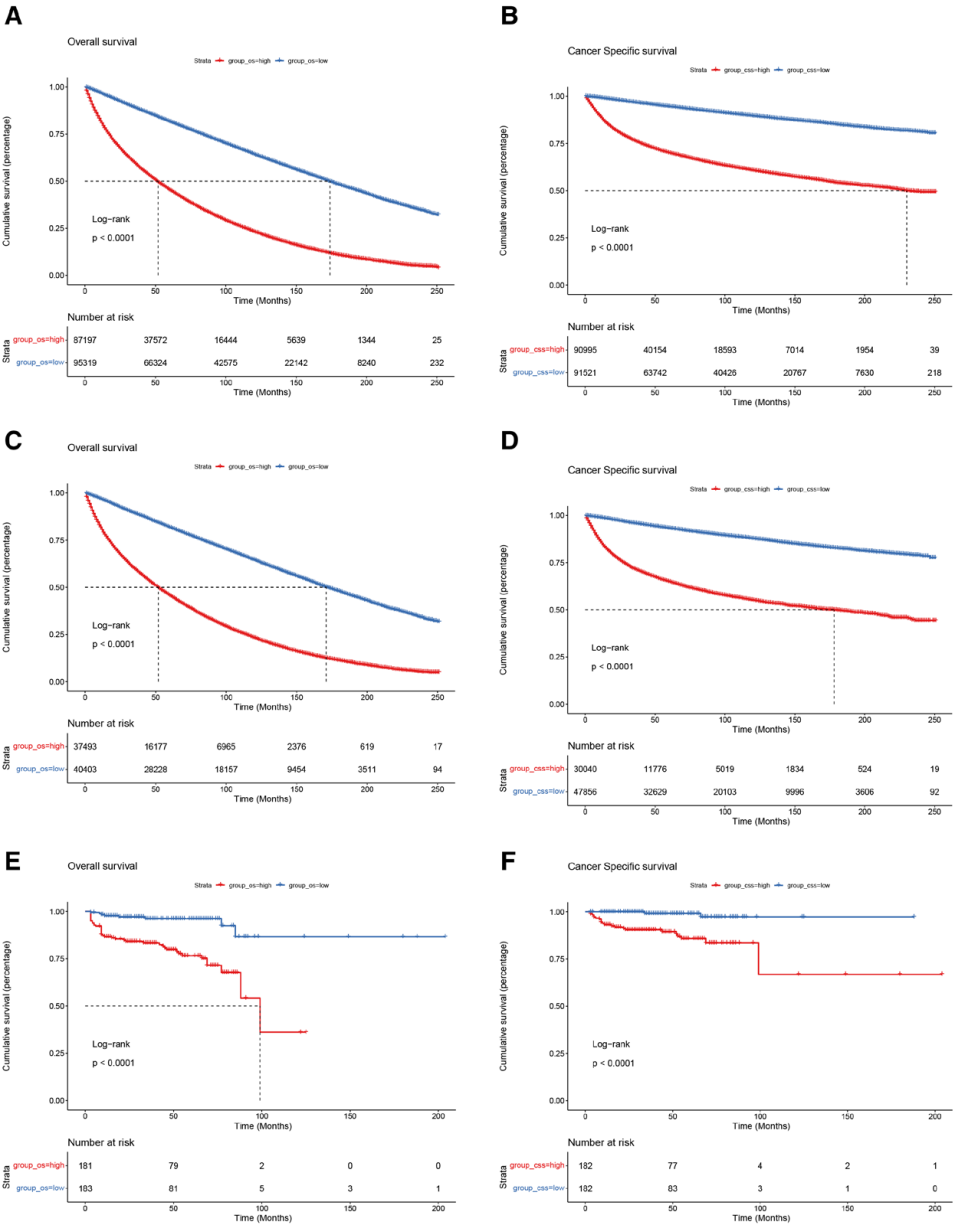
Kaplan–Meier curves of patients in the low-risk and high-risk groups. The K-M curve showed that the OS rate of the patients in the high-risk group was significantly lower than that in the low-risk group in the training (A), internal validation (C) and external validation (E) cohorts. The K-M curve showed that the CSS rate of the patients in the high-risk group was significantly lower than that in the low-risk group in the training (B), internal validation (D) and external validation (F) cohorts. CSS = cancer specific survival, OS = overall survival.

**Figure 8.**
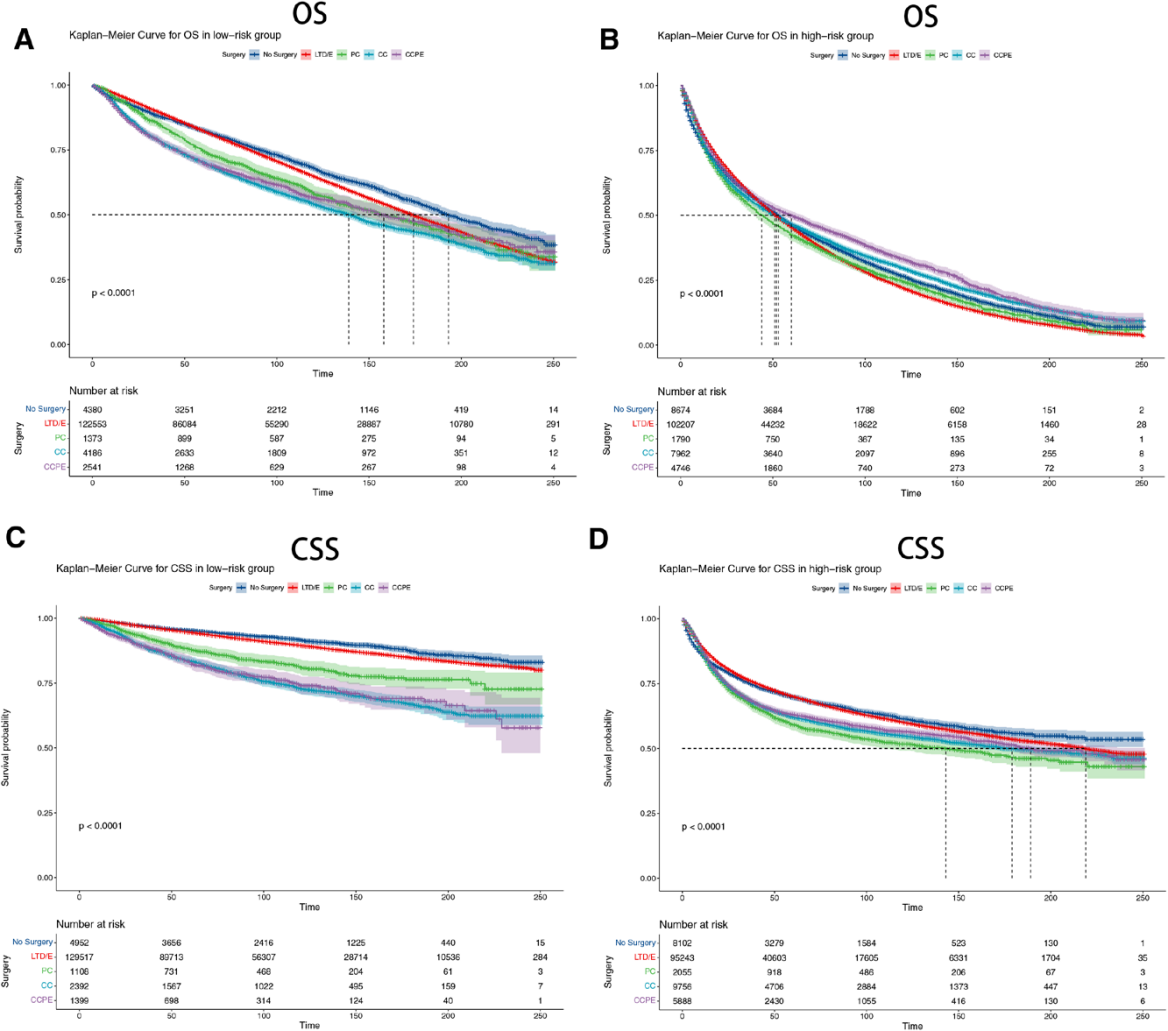
Kaplan–Meier curves of patients treated by different surgery. (A) The OS rate of patients in the low-risk group underwent different surgery. (B) The OS rate of patients in the high-risk group underwent different surgery. (C) The CSS rate of patients in the low-risk group underwent different surgery. (D) The CSS rate of patients in the high-risk group underwent different surgery. CC = complete cystectomy, CCPE = complete cystectomy with pelvic exenteration, CSS = cancer specific survival, LTD/E = local tumor destruction/excision, OS = overall survival, PC = partial cystectomy.

In the training, internal validation and external validation cohorts, the DCA indicated that the clinical application value of the nomograms predicting OS and CSS is higher than T stage (Fig. 6). Based on the best cutoff value, we allocated all patients to the high-risk group (total score ≥ 114.69) and the low-risk group (total score < 114.69) in terms of OS, and we classified all patients into the high-risk group (total score ≥ 159.8) and the low-risk group (total score < 159.8) in terms of CSS. In both training and validation cohorts, the K-M curves displayed that non-metastatic BC patients in the low-risk group owned significantly higher OS and CSS than patients in the high-risk group (*P* < .001) (Fig. 7). In the high-risk group, the 3-, 5-, and 8-year OS rates of patients were 59.15%, 45.36%, and 30.46%, respectively. In the low-risk group, the 3-, 5-, and 8-year OS rates of patients were 88.8%, 81.6%, and 71.1%, respectively. In the high-risk group, the 3-, 5-, and 8-year CSS rates of patients were 76.2%, 70.0%, and 63.9%, respectively. In the low-risk group, the 3-, 5-, and 8-year CSS rates were 96.7%, 94.6%, and 91.6%, respectively. In the high-risk group, the K-M curves indicated that patients treated by partial cystectomy displayed significantly lower CSS (*P* < .001), while patients underwent complete cystectomy with pelvic exenteration displayed significantly higher OS (*P* < .001) (Fig. 8). And in the low-risk group, patients underwent complete cystectomy displayed significantly lower OS and CSS (*P* < .001), while patients with no surgery displayed significantly higher OS and CSS (*P* < .001) (Fig. 8).

### 3.5. Online application for survival prediction

We have constructed a user-friendly online application for clinicians to predict the OS and CSS of non-metastatic BC patients based on our nomograms. The website for OS is https://lishan123.shinyapps.io/DynNomapp/, and the website for CSS is https://lishancss.shinyapps.io/DynNomapp/. Clinicians can easily obtain estimated OS and CSS probability after inputting patient characteristics in our nomograms, getting convenience for predicting prognosis and tailoring treatment plan in clinical practice.

## 4. Discussion

BC makes up approximately 500,000 new cases and 200,000 deaths around the world, and there are over 82,000 new cases and 16,000 deaths every year in the United States.^[1,19]^ BC constitutes presumably 90% to 95% of urothelial cancer,^[20]^ which featured in “umbrella” cells that line the lumen of the urinary bladder. Theoretically, urothelial cancers involve tumors of the bladder, upper urinary tract (renal pelvis and ureters), and proximal urethra. In addition, several molecular and genetic subtypes of BC have been discovered based on comprehensive profiling efforts, such as The Cancer Genome Atlas (TCGA) project.^[21]^ Nonetheless, these subtypes are not extensively applied in clinical practice due to inadequate evidence supporting their prognostic and predictive value. As far as we are concerned, no previous study has constructed and validated nomograms to forecast OS and CSS of non-metastatic BC patients. Hence, we attempted to build a precise prognostic model to help clinicians effectively assess the patient prognosis and make treatment decisions. We included 260,412 non-metastatic BC patients from the SEER database, and we enrolled 364 non-metastatic BC patients from The First Affiliated Hospital of Chongqing Medical University as an external validation cohort. Finally, we succeeded in developing nomograms to forecast 3-, 5-, and 8-year OS and CSS of non-metastatic BC patients, while internal and external validation showed favorable accuracy and discrimination.

As shown in nomograms, age is an influential prognostic factor for non-metastatic BC patients. Traditionally, age is considered the most powerful risk factor for BC patients, who are diagnosed between 70 and 84 years on average.^[22]^ Epidemiologists showed that BC patients are rarely under 50 years,^[23]^ which may be owing to an age-related decreased ability to repair DNA and endure treatment-induced toxicity.^[24,25]^ As for sex, it seems that gender has little effect in the nomograms, which predicts that male patients have worse OS than female patients. It is 3 to 4 times more frequent for men to be diagnosed as BC than women, usually due to exposures, lifestyle, and stasis of urine-containing carcinogens in men with prostatic enlargement and urinary retention.^[24,26]^ However, in a prior study controlling smoking and occupational hazards, the disparity in sex-related risk of BC remained.^[27]^ Similarly, a previous meta-analysis concluded that the sex prevalence of smoking only partially explains the sex difference in BC incidence.^[28]^ Researchers determined that the sex difference in BC incidence is not associated with differences in exposure risk including smoking status.^[26]^ Moreover, some researchers have drawn a similar conclusion that female BC patients may have worse prognosis,^[29,30]^ and it is reported that advanced BC is more prevalent in women than men.^[31,32]^ Hematuria in women is usually ascribed to infection, causing delayed diagnosis of BC in women.^[26]^ Researchers have suggested that the role of hormone receptors and genomic differences in female patients may partially account for survival differences of sex.^[33]^

Cox regression analysis showed that black patients owned worse OS and CSS, which is consistent with the previous discovery that African Americans have worse disease-specific outcomes and higher proportions of unfavorable pathology.^[11,34]^ Different genetic characteristics, molecular markers, and lifestyles between races probably account for this disparity, which still needs to be further investigated. The models display that married patients have best OS and CSS, which can be explained by mechanisms of social support. Married patients are likely to own more financial resources, get more social support, enjoy a higher life quality, live a healthier lifestyle, and undergo better treatment than unmarried or widowed patients.^[35–37]^

As for location of the tumor, we discovered that tumors with overlapping lesions display the worst prognosis. And tumors located in bladder base have worse OS and CSS than that located in lateral wall of bladder, while tumors located in urachus or dome display the best prognosis. Our conclusion is consistent with a previous study based on the SEER database,^[38]^ which can be explained by the anatomical and histological features of bladder base. The bladder neck is fixed and located at the lowest point of the bladder, which is different from other muscular tissues in terms of tissue, with smaller smooth muscle cells and a tighter distribution structure of intercellular and connective tissues.^[39]^ This anatomical feature may lead to increased incidence and recurrence of tumors.^[40]^ Meanwhile, an important feature of urothelial carcinoma (transitional cell carcinoma) is the simultaneous or sequential development of multiple lesions throughout the urinary tract, which indicates that the risk of concurrency with other tumors is depended on the primary location of the tumor. It is reported that BC originating in bladder trigone is six times more possible to develop tumors of upper urinary tract than other BC,^[41]^ and BC located in trigone and neck of the bladder should be considered high-risk markers for developing prostate cancer.^[42]^

BC is a highly heterogeneous disease entity, over 90% of BC patients are diagnosed with urothelial carcinoma, with the rest having squamous cell carcinoma, adenocarcinoma, or neuroendocrine tumor.^[43]^ Most bladder tumors with histological variants are diagnosed at advanced stages with extravesical disease and metastasis.^[44]^ In nomograms, we discovered that patients diagnosed with transitional cell carcinoma have the highest OS and CSS. Grade is clinically relevant to the patient prognosis, but different clinical guidelines recommend different pathological reports according to the 1973 or 2004/2016 WHO grading systems, with G1-G3 or low-grade (LG), high-grade (HG) and the category of papillary urothelial neoplasms of low malignant potential (PUNLMP), which may bias our analysis.^[45]^ This may explain why patients at grade III and patients at grade IV rank similarly in nomograms. T stage has great weight in our models. It is reported that the depth of bladder invasion significantly influenced the prognosis, given that tumors with more advanced T stage are more aggressive and progressive.^[46–48]^ Meanwhile, tumor size was considered an independent prognostic factor for OS in BC patients after RC.^[49]^ In our study, we also concluded that the tumor size is inversely proportional to the prognosis of non-metastatic BC patients.

Multimodal treatments including surgery, chemotherapy, and radiotherapy are the main therapy methods to treat BC patients.^[50,51]^ In our study, patients underwent surgery had higher OS and CSS than those who did not receive surgery, which can be explained by the consensus that most non-metastatic bladder tumors are resectable.^[50,52]^ Chemotherapy was considered a vital treatment option for BC patients, and the prognosis of those not treated with cisplatin is poorer than those treated with carboplatin.^[53]^ When suspecting low- and intermediate-risk disease, intravesical chemotherapy should be implemented within 24 hours after TURBT to kill free-floating tumor cells, thus mitigating seeding of the urothelium.^[51]^ Neoadjuvant chemotherapy before RC is recommended in AUA and EAU guidelines.^[50]^ In our study, patients who receive chemotherapy have higher OS and CSS than those not received chemotherapy. And Cox regression analysis discovered that patients receiving neoadjuvant chemotherapy owned a better prognosis than other types of chemotherapy. Meanwhile, patients who receive radiotherapy have lower OS and CSS than those not received radiotherapy. But patients received radiotherapy before and after surgery owned better prognosis than other type of radiotherapy. Adjuvant radiotherapy still needs investigation to determine its effects.^[44]^ A multicenter randomized controlled trial including 210 patients with T1NxM0 stage and Grade III revealed no statistical difference in 5-year OS, progression-free survival, and recurrence-free survival between the radiotherapy group and the control group.^[54]^ Lymph node dissection is helpful for predicting prognosis and administering adjuvant therapy, as 25% and 8% of MIBC and high-risk NMIBC patients present lymph node metastases at the time of RC.^[20]^ Lymph node dissection is beneficial for BC patients, as about 20% of patients with positive lymph nodes obtain better prognosis after lymph node dissection.^[55]^ We found that patients underwent dissection of lymph nodes have a better prognosis, which agrees with the previous conclusion that increased lymph node harvest could provide oncological benefits in BC patients.^[56]^

Nevertheless, our study has several limitations. Firstly, our research is a retrospective study, resulting in a possibility for selection bias. Secondly, although we enrolled an external validation cohort in Chongqing, some variables are not available in the SEER database and our cohort, such as smoking, drinking, BMI index, occupational hazards, comorbidities, and genetic factors which probably makes our models incomprehensive.^[57–59]^ But we yet incorporated key variables and proved a good accuracy, so there would not be a devastating deviation. Thirdly, progression-free survival and recurrence-free survival which cannot be calculated from the SEER database are vital for assessing the prognosis, and therapies applied due to a tumor relapse will partly affect OS and CSS. Last but not least, chemotherapy and radiotherapy, as general term in nomograms, require clarification about adjuvant and neoadjuvant, which is mostly unknown in the SEER database. Owing to the missing data, we cannot conduct a more precise model to forecast the patient prognosis.

## 5. Conclusion

We establish novel nomograms to forecast the OS and CSS in non-metastatic BC patients, which displayed better predictive capability than current TNM staging system. The models are internally and externally validated with favorable precision and discrimination. And the exploration of prognostic factors and the establishment of nomograms will assist clinicians to evaluate the patient prognosis accurately and make good clinical decisions.

## Acknowledgments

We are grateful to the Surveillance, Epidemiology, and End Results (SEER) database for providing data.

## Author contributions

**Conceptualization:** Shan Li, Jinkui Wang, Junhong Liu, Dawei He.

**Data curation:** Shan Li, Jinkui Wang, Zhaoxia Zhang, Yuzhou Wu, Zhenyu Liu.

**Formal analysis:** Shan Li, Jinkui Wang, Zhaoxia Zhang.

**Funding acquisition:** Jinkui Wang, Zhikang Yin, Junhong Liu, Dawei He.

**Investigation:** Shan Li, Zhaoxia Zhang.

**Methodology:** Shan Li, Zhaoxia Zhang, Dawei He.

**Supervision:** Zhikang Yin, Junhong Liu, Dawei He.

**Validation:** Shan Li, Jinkui Wang.

**Visualization:** Shan Li, Jinkui Wang.

**Writing – original draft:** Shan Li.

**Writing – review & editing:** Zhikang Yin, Junhong Liu, Dawei He.

## Supplementary Material


