# Supplementary material for: Establishment and validation of nomograms to predict the overall survival and cancer-specific survival for non-metastatic bladder cancer patients: A large population-based cohort study and external validation
Source: Medicine (Baltimore). 2024 Mar 15;103(11):e37492. doi: 10.1097/MD.0000000000037492 (PMC10939645; doi:10.1097/MD.0000000000037492)
Supplement: Supplementary file 1 [file medi-103-e37492-s001.docx]

**Supplementary Table 1** Clinicopathological characteristics of non-metastatic BC patients in the external validation cohort from The First Affiliated Hospital of Chongqing Medical University. BC: bladder cancer.

|  | Overall N=364 | Alive N=314 | Dead N=50 | p.overall |
| --- | --- | --- | --- | --- |
| Age: |  |  |  | 0.601 |
| <40 | 11 (3.02%) | 11 (3.50%) | 0 (0.00%) |  |
| 40-59 | 103 (28.3%) | 88 (28.0%) | 15 (30.0%) |  |
| 60-79 | 216 (59.3%) | 187 (59.6%) | 29 (58.0%) |  |
| >=80 | 34 (9.34%) | 28 (8.92%) | 6 (12.0%) |  |
| Age (continuous): | 64.7 (11.9) | 64.5 (12.1) | 65.9 (10.9) | 0.393 |
| Race: Other | 364 (100%) | 314 (100%) | 50 (100%) | . |
| Sex: |  |  |  | 0.009 |
| Male | 288 (79.1%) | 241 (76.8%) | 47 (94.0%) |  |
| Female | 76 (20.9%) | 73 (23.2%) | 3 (6.00%) |  |
| Marital Status: |  |  |  | 0.256 |
| Married | 362 (99.5%) | 313 (99.7%) | 49 (98.0%) |  |
| Widowed | 2 (0.55%) | 1 (0.32%) | 1 (2.00%) |  |
| Household Location: |  |  |  | 0.708 |
| Rural | 16 (4.40%) | 15 (4.78%) | 1 (2.00%) |  |
| Urban | 348 (95.6%) | 299 (95.2%) | 49 (98.0%) |  |
| Tumor Primary Site: |  |  |  | 0.246 |
| Bladder wall | 253 (69.5%) | 212 (67.5%) | 41 (82.0%) |  |
| Bladder base | 96 (26.4%) | 88 (28.0%) | 8 (16.0%) |  |
| Urachus/Dome of bladder | 11 (3.02%) | 10 (3.18%) | 1 (2.00%) |  |
| Overlapping lesion | 4 (1.10%) | 4 (1.27%) | 0 (0.00%) |  |
| Histology: |  |  |  | 0.246 |
| Adenocarcinoma | 3 (0.82%) | 2 (0.64%) | 1 (2.00%) |  |
| Other | 3 (0.82%) | 2 (0.64%) | 1 (2.00%) |  |
| Squamous cell carcinoma | 6 (1.65%) | 5 (1.59%) | 1 (2.00%) |  |
| Transitional cell carcinoma | 352 (96.7%) | 305 (97.1%) | 47 (94.0%) |  |
| Grade: |  |  |  | <0.001 |
| I | 221 (60.7%) | 202 (64.3%) | 19 (38.0%) |  |
| II | 101 (27.7%) | 85 (27.1%) | 16 (32.0%) |  |
| III | 38 (10.4%) | 25 (7.96%) | 13 (26.0%) |  |
| IV | 4 (1.10%) | 2 (0.64%) | 2 (4.00%) |  |
| Laterality: |  |  |  | 0.524 |
| Bilateral | 5 (1.37%) | 4 (1.27%) | 1 (2.00%) |  |
| Lateral | 359 (98.6%) | 310 (98.7%) | 49 (98.0%) |  |
| T Stage: |  |  |  | <0.001 |
| T1/Tis/Ta | 227 (62.4%) | 206 (65.6%) | 21 (42.0%) |  |
| T2 | 98 (26.9%) | 83 (26.4%) | 15 (30.0%) |  |
| T3 | 27 (7.42%) | 20 (6.37%) | 7 (14.0%) |  |
| T4 | 12 (3.30%) | 5 (1.59%) | 7 (14.0%) |  |
| Tumor Size: |  |  |  | 0.030 |
| 0-2.0 | 213 (58.5%) | 190 (60.5%) | 23 (46.0%) |  |
| 2.1-4.0 | 122 (33.5%) | 104 (33.1%) | 18 (36.0%) |  |
| 4.1-6.0 | 19 (5.22%) | 13 (4.14%) | 6 (12.0%) |  |
| 6.1-8.0 | 8 (2.20%) | 5 (1.59%) | 3 (6.00%) |  |
| >10.0 | 2 (0.55%) | 2 (0.64%) | 0 (0.00%) |  |
| Surgery Type: |  |  |  | 0.002 |
| No Surgery | 5 (1.37%) | 3 (0.96%) | 2 (4.00%) |  |
| Local tumor destruction/excision | 227 (62.4%) | 204 (65.0%) | 23 (46.0%) |  |
| Partial cystectomy | 17 (4.67%) | 17 (5.41%) | 0 (0.00%) |  |
| Complete cystectomy | 111 (30.5%) | 88 (28.0%) | 23 (46.0%) |  |
| Complete cystectomy with pelvic exenteration | 4 (1.10%) | 2 (0.64%) | 2 (4.00%) |  |
| Surgery Other Sites: |  |  |  | 0.031 |
| None | 235 (64.6%) | 210 (66.9%) | 25 (50.0%) |  |
| Yes | 129 (35.4%) | 104 (33.1%) | 25 (50.0%) |  |
| Lymph Nodes Surgery: |  |  |  | 0.044 |
| None | 232 (63.7%) | 207 (65.9%) | 25 (50.0%) |  |
| Regional lymph nodes removed | 132 (36.3%) | 107 (34.1%) | 25 (50.0%) |  |
| Chemotherapy: |  |  |  | 0.099 |
| None/Unknown | 93 (25.5%) | 75 (23.9%) | 18 (36.0%) |  |
| Yes | 271 (74.5%) | 239 (76.1%) | 32 (64.0%) |  |
| Radiation: |  |  |  | 1.000 |
| None/Unknown | 362 (99.5%) | 312 (99.4%) | 50 (100%) |  |
| Yes | 2 (0.55%) | 2 (0.64%) | 0 (0.00%) |  |
| Neoadjuvant or Adjuvant Chemotherapy: |  |  |  | 0.283 |
| None | 91 (25.0%) | 73 (23.2%) | 18 (36.0%) |  |
| chemotherapy before surgery | 2 (0.55%) | 2 (0.64%) | 0 (0.00%) |  |
| chemotherapy after surgery | 258 (70.9%) | 227 (72.3%) | 31 (62.0%) |  |
| before and after | 13 (3.57%) | 12 (3.82%) | 1 (2.00%) |  |
| Neoadjuvant or Adjuvant Radiotherapy: |  |  |  | 1.000 |
| None | 362 (99.5%) | 312 (99.4%) | 50 (100%) |  |
| radiation after surgery | 2 (0.55%) | 2 (0.64%) | 0 (0.00%) |  |
| Survival Months | 45.4 (30.1) | 48.4 (29.4) | 26.0 (27.8) | <0.001 |
| Cancer Specific Death: |  |  |  | <0.001 |
| Not cancer specific death | 340 (93.4%) | 314 (100%) | 26 (52.0%) |  |
| Dead due to bladder cancer | 24 (6.59%) | 0 (0.00%) | 24 (48.0%) |  |
